# Résumé of Work on “Epileptiform Syphilis”

**Published:** 1880-05

**Authors:** Celso Pellizari

**Affiliations:** Florence


### ARTICLE IV.

RÉSUMÉ OF WORK ON "EPILEPTIFORM SYPHILIS." By DR. CELSO PELLIZARI, of Florence.

In the first place I propose to call "epileptiform syphilis" those complicated phenomena which produce that form of convulsions which, until now, have been designated under the names of "hemiplegic epilepsy," "epileptic hemiplegia," "muscular spasms." Most all the authors have agreed to comprehend under the name of "syphilitic epilepsy" all the convulsive forms which depend on syphilis. Others, after the denomination of "spasms muscular," given by Tach to these convulsive phenomena, have said that the *tout ensemble* of phenomena did not deserve the name of epilepsy, as there were lacking some of its characteristics. The names of "hemiplegic epilepsy," "partial epilepsy," "epileptic hemiplegia," "hemi epilepsy," etc., comprehend only particular forms, and therefore cannot comprehend them all. As these convulsions manifest themselves always with the form of attack which gives an immediate idea of the epileptic fit, I have proposed the name of epileptiform syphilis, which, in my judgment, is better suited to the said disease, as whatever may be the form, the attack is always there, and gives at once the idea of epilepsy.

Case 1. A man 38 years old was admitted in the hospital of San Maria Nova, on the 24th of May, 1878, on account of convulsions, which had not left him for some days. He had had syphilis 16 years before, and bore marks on his body which were, beyond any doubt, those left by syphilitic sores. As to the convulsions, he had had the first in July, 1877, while at work. Taken to the hospital then, they had applied ice upon his head, and the attack did not return. From that time until the 20th of May, 1878, the patient had no more convulsions, only he complained of general weakness and dizziness. The 20th of May he was taken by dizziness, which continued till the 22d. In the night of the 22d to the 23d he had two convulsive attacks. In the following night the convulsions occurred five or six times; on the 24th the attacks became more numerous, and the patient could give no account of himself whatever. The convulsions had always the same form, and were *unilateral.* The attack was in form of partial epilepsy. The patient began to open the eyelids and roll his eyes towards the left. The left angle of the mouth was strongly drawn one side; therefore all the left side of the face felt the convulsions almost contemporaneously; the patient extended the left arm, closed the fingers, and the thumb remained pressed between the fore finger and the third finger; the cyanosis succeeded very soon, and the convulsion was at an end. During the attack there was involuntary emission of urine and of fæces. During the first night of his stay in the hospital the patient grew considerably worse; the next day (25th) continued to have the attacks stronger, and more and more frequently. After the convulsion he went into a profound drowsiness, from which it was impossible to bring him out. Treatment with the frictions of (ʒi) 4 grams of the mercurial salve, and a potion with (grs. xlv) 3 grams of iodide of potassium was begun at once. Leeches were applied, and also a bag of ice which was placed on his head permanently; however, all was useless, and the patient died at 10 a. m. on the 26th. The convulsions had grown worse in frequency and intensity; an augmentation of temperature was visible during the last 30 hours, but until then had been natural all the time. The diagnosis was that we had to deal with a gummy pachymeningitis of the excito-motrix zona of the right, admitting, however, that an exostosis, or a gumma of the cortical substance could have given the same phenomena; let us add, besides, that probably to the specific form was added an acute attack of inflammation, which alone could account for the hasty death of the patient. In fact, at the autopsy the dura mater was found stretched, and the meningea media dilated with liquid blood. Against the right frontal lobe of the brain, very near the orbitar plane, the dura meningia was soldered with the cerebral substance to the extent of the size of a piece of 5 francs. The adhesions were so tenacious that they could not be detached without a knife; in the middle of the adhesions were found, here and there, small bony and calcareous formations. The cortex of the brain in this spot was, to a great extent, destroyed, and its place taken by well-organized connective tissue. In the spaces under the arachnoid was infiltrated a small quantity of clear serum. The pia mater di not peel nicely from the convolutions. On the left, between the second and third frontal convolutions were noticed two small osteomata, and in the middle of the second convolution a small nodule as brilliant as a raw tubercle, which made a slight protuberance on the surface. The nervous cerebral substance appeared a little more consistent than usual; in the annular protuberance was a grayish spot, occupying the inferior segment of the right cavity. The veins were swollen and full of blood; however, the vessels did not show any alteration of their walls. Besides these alterations, there was disease of the tibia, and gummy nodules of both testicles.

In my pamphlet I have talked at considerable length upon certain considerations, in order to prove that there is not another disease besides syphilis which could present such a clinical and anatomical table. Then I gave my second observation, as follows: A boy aged 8, born of syphilitic parents, who had himself been under treatment at various times for congenital syphilis. This boy was taken to the hospital of S. M. Nova on the 26th of November, 1878, on account of convulsions, which had occurred daily for about ten days, but only affected the left side of the body. The specific treatment, with mercurial frictions and iodide of potassium internally, gave great improvement; the fits became much less frequent, and the child began to walk about the ward. All was apparently going well, when the patient was suddenly taken with typhoid fever, and died in a few days, that is to say, on the 28th of December.

*Post Mortem:* The skull and dura mater were found quite healthy, as was also the pia mater, except opposite the right paracentral lobe, where it was a little thick and opaque. There was slight wasting of the brain substance. No gumma nor tubercles were found. At the upper part of the right lung was a circumscribed patch of pneumonia, surrounding a cicatrix, which seemed to be that of a by-gone gumma. The liver presented a deeper cicatrix on its convex surface. In the intestines, clear evidence of typhoid fever was found in Peyer's glands. Taking into consideration the ideas of well-informed writers, I believe that the slight alterations, found at the autopsy, are not without some worth. In fact, the autopsy only showed what the clinic had declared it to be; that is, the child cured of convulsions, and dying afterwards of another disease. As the treatment was successful in making the convulsions disappear, it ought, also, to have destroyed the material cause, as well.

My third observation is a man of 36 years. He entered the syphilitic clinic of Florence on the 5th of February, 1879. His mother had suffered from convulsions of epileptic form for twelve years, which had begun with the fourth confinement. The patient was born during the interval of these twelve years, but all his youth was passed in perfect health. He had been treated in the said clinic for syphilis by Prof. Pietro Pelizzari (my uncle) in 1863; in 1877 he began to have pains in the head, which did not have a very decided nature. Shortly after the pains had begun, a kind of ulcer showed itself on the inferior lip, for which he was ordered alteratives. In three months the sore was cured, but the pains, although weaker, continued. In this condition he went till March, 1878. The 7th of the month he began to stammer and feel faint, without, however, losing consciousness. Ten minutes later he was taken by a convulsive attack. In the same day he had another attack, and it was reported, with loss of consciousness. They gave him bromide of potassium and nux-vomica, but the convulsions continued with phenomena of amnesia. The convulsive fits were always partial, only the right part of the body entered into convulsions. The fits, which were always preceded by stammering, and which did not deprive him of consciousness, had intervals of fifteen to twenty days, but when they occurred, they were more numerous. In October, having had stronger fits, and some tubercles having appeared in groups and ulcerated on the right thigh, he went to Prof. P. Pelizzari, who, determining it to be a specific cerebral affection, ordered the treatment, with the frictions of (ʒj) 4 grams of mercurial salve, and iodide of potassium, from (℈j–ʒss) 1 gram to 2½ per day. Under this treatment the sores disappeared and he was free from convulsions for two months. Towards the end of December the convulsions reappeared (he then came to Florence, when I saw him for the first time), but with these a weakness of intellect and of muscular power was visible; there was paresis of the right arteries and a slight deviation of the tongue towards the right. The iodide of potassium helped him considerably, and put a stop to the convulsions. On the evening of February 2, 1879, having drunk of wine and smoked too much, the patient was again taken with convulsions. The fits, which at first occurred every hour, grew so numerous that, on the 4th, when I was called in to see him, they did not leave him ten minutes at a time. The fits, which lasted one minute or a little more, were still limited to the right half. The patient was quite prostrated. The 5th of February, when taken to the hospital, he was in such a condition that it was impossible to get one word out of him, and the attack had lost its unilateral type. There was no fever. The diagnosis of the clinic was that of meningitis-gummosa in the left occipital zone. The treatment was: Frictions with mercurial salve, (ʒj) 4 grams; iodide of potassium, (ʒj) 4 grams. Applications of ice upon the head permanently. Having obtained no improvement, the following day the ice was taken off, in order to make a revulsion by applying a blister on the neck and sinapisms to the inferior extremities. Under this treatment there was a slight improvement, which lasted about two days. On the morning of the 10th he was much worse; was taken with strong fever, the convulsions increased in frequency, and were general. Death occurred on the 13th of February, in the evening.

*Post Mortem:* The dura mater, on the left, appeared more opaque than on the right, although at that side there was a spot where it was thicker. Nothing morbid was found on the right. On the left nothing morbid appeared corresponding to the occipital lobe, but towards the frontal lobe the dura mater was found adhering to the under membrane, beginning at Rolando's furrow. These adhesions were not strong for a short space, but in correspondence with the frontal convolutions, were so tenacious that it was impossible to detach the dura mater without lacerating the nervous substance. These adhesions were found to correspond to the first, second, and third frontal convolutions, beginning from their orbitar extremity, and existed even upon the inter-hemispheric side of the first frontal. However, the posterior extremity of the third frontal, or convolution of Broca, was not included in the area of the tenacious adhesions. In correspondence with the same, and with the ascending frontal, the adhesions could be broken with the finger without laceration of the cerebral substance. The nervous substance of the left frontal lobe was softened, diffluent and white. The softening destroyed the cortical substance and the oval center corresponding to all the first and second frontal, and of the anterior two-thirds of the third, deepened to the external capsule, which seemed intact to the naked eye. Near the base of the third frontal, and of the ascending frontal, the pia mater detached itself with difficulty from the cerebral surface, and there the cortical substance was of a pinkish lavender. The cerebral arteries were sound. No alteration of the viscera of the chest, nor of the abdomen. Although the mother had conceived him while she was epileptic, it was difficult to believe that he was affected by hereditary epilepsy. In fact, in this case, the convulsions would have been determined when he developed, but not that special form, nor with these alterations. I believe firmly that the patient died of cerebral meningitis, which was due to syphilis, and I don't think it posssible to attribute it either to alcoholism or to a thrombus.

In the second part of my work, I speak of epileptiform syphilis in general. As to the etiology, I believe that epileptiform syphilis develops itself rather more in those syphilitics who have an hereditary disposition, but, among the other causes which help in developing that disease, are wounds of all kinds, abuse of mental fatigue, alcohols, and "pleasures of Venus," as the Italians term it. That form of cerebral syphilis may occur at all ages. Le Pileur tells of a case of epileptic convulsions from syphilis in a child one month old. In our three cases we find a patient of eight, and another of thirty-eight. The time which elapses from the syphilitic infection to the determination of the fits, varies from some months to sixteen years, in the cases known till now. I do not think that it is possible yet to determine whether this form of convulsions comes sooner in those who have not used mercury, or in those who have abused it, or those who have used it moderately. The statistics in medicine are not precise enough. The anatomic alterations, which generally cause the epileptic form are several. The alteration of the bones of the cranium may determine the different convulsive forms, either directly, with exostosis, which exercises compression upon the cortical, or indirectly, when they are caused by meningeal affections. The gummy form of the meninges is admitted by every one to be the most common alteration in this disease ; it may be circumscribed to a tumor or diffused. The disease of the brain, and in particular of the cortex, may even cause the convulsions. Finally, as to the parts which have the essential alterations, in this disease, there is still some doubt ; some authors admit that even the alteration of the vessels alone may occasion epileptiform syphilis (I speak of it in my work on cerebral disorders). I don't believe the following question well answered yet, that is to say : "Where are to be found the lesions which may give origin to the epileptic convulsions ?" I am rather inclined to admit, with Charcot, that in partial epilepsy, the alteration ought to reside at the surface of the frontal convolution and *ascending parietal*, or, at least, in their immediate vicinity. Are these convulsive fits always the expression of a material lesion, or is there also an epileptiform syphilis *sine materiâ ?* I believe that conclusive observations upon epileptiform syphilis, in which the autopsy has not shown any alteration, *do not exist ;* but I believe that alteration of the blood and of the lymphatic system when infected can have the power in certain persons and in special conditions to give place to convulsive fits of itself. I do not deny epileptiform syphilis *sine materiâ*, but I want it restricted to a very few cases. At all events, these discrasic forms cannot correspond absolutely to the common one. They must have a great variability of *seat* and of form, and they must be quite brief; in these the fits will not repeat themselves with so much persistence, will not have so significant a form, and will not be so limited to any one part of the body, and will never be followed by paralytic phenomena, which persist after the convulsions. The only protean forms of specific epilepsy which may have value in practice in preventing a fatal result, are the cephalalgiæ, which, although they do not always take place, occur in the greater part of cases, and sometimes appear many months before the convulsive attack. Their characteristics would be persistent, and localized in the forehead. The character of the fits is not always the same; many times they are preceded by something out of the ordinary course, such as a fixed pain in a finger, a feeling of pricking like stinging of ants, of noise in the ears, of trouble in the eyes, of chills, muscular trepidation, of gasping, etc., which give the patient to understand the convulsion is coming. It may be general or partial. It is, however, oftener partial than general, and also, in the cases in which the convulsions become general, the prevalence in one part still remains. The length of time that the attacks last is much varied, as also is their number. The entire loss of consciousness occurs seldom with these fits. Among the morbid remains of the convulsions are noted: fatigue and deep prostration felt by the patient; paralysis, alteration of the sensibility, and finally intellectual disturbances, which may oscillate from simple diminution of the memory to dementia. The diagnosis of epileptiform syphilis is pretty difficult, and on account of it, the physician must not content himself with studying the attack as long as it has no special patronymic symptoms, but he will have to notice very carefully the other syphilitic alterations which may accompany it, the age of the patient, and, above all, work with zeal and patience to find out and put in evidence the pre-existence of syphilis. Another most important item is the ensemble of phenomena presented by the patient rather than that of a separate symptom; that is to say, of the convulsions, the form in which they develop themselves, the way in which they show themselves, and the prodromata which accompany or follow them. Finally, the physician may also be helped by the therapeutic judgment, which may be had, also, when we see the patient for the first time, when he has suffered beforehand from the common phenomena which precede the convulsions, as, for example, cephalalgiæ, dizziness, etc., and has been cured of them by specific treatment. The prognosis is serious, however its dangerous nature may vary, according to the alterations which are supposed to have caused these convulsions, and according, also, to the time that the sickness lasted, since, in the greater number of cases, the short duration of the symptoms indicates relatively the slight intensity of the material alteration. As to the cure, I beg my readers to observe that it is not as easy as it might seem to be. After having fought the ideas of those who admit the tardy forms due to the mercury, instead of to the syphilis, I do not deny that the remedy, taken in exaggerated doses, or in certain organisms, may produce some trouble in the cerebral tissues. In general, I do not approve of the energetic mercurial treatment for slight phenomena, whether of the skin or of another system; I neither approve of too prolonged treatment. I prefer to do it little by little, at intervals, giving, for example, mercurial preparations for ten days, and for the ten following days, iodide of potassium; after the last, leaving the patient for a week or two without any medicine, and this in order to prevent the accumulation of mercury in the organism. This is also the method I prefer for cerebral phenomena which have shown themselves for some time but slightly. Of course I know this ideal treatment cannot be put in practice in cerebral syphilis, but, occasionally, with a patient who has attacks daily, without intermission, the treatment must be made with the most powerful means; frictions with (ʒj) 4 to 5 grams of mercurial salve, or with hypodermic injections, united with the iodide of potassium, in a dose of (ʒss–j) 2, 3 to 4 grams, and sometimes more, daily. There may be, also, some difference in the cases where it is necessary to act energetically. For example, in the time nearer infection, it would be necessary to confide more in mercury than in the iodide of potassium; but the contrary, when the patient has used mercury freely and a good deal in a short time. In cases where there is supposed to be an exostosis by condensing osteitis, as of the fibroid form of the meninges, or of the tunics of the vessels, it will be necessary to use mercury sparingly, and give, on the contrary, much importance to iodide of potassium prolonged. The most energetic mercurial and iodide treatment will be made, when there will be reason to think of the real gummy form, especially in the cerebral substance. Certainly it is very difficult to say, if, to the specific alteration, there be added a simple inflammation or a softening; but when the diagnosis can be made, it is the duty of the physician not to insist on mercurials. In this case I believe that an accumulation of mercury in an organism may contribute largely to accelerate death. It has been noted how, in epileptiform syphilis, the iodide of potassium, after some time, had no more effect, and that bromide has been an excellent substitute. Finally, the physician must recollect that, besides the remedies called specifics, there are others which may be required by the acute and decidedly phlogistic phenomena, by the severe reflex irritation, by the state of drowsiness, by the posture, etc., which may have occurred, and these remedies are: ice, bleeding, sedatives, excitants, hydrotherapy and electrotherapy.

